# Assessment of pain neurophysiology knowledge among healthcare students in Bayero University Kano, Nigeria: a cross-sectional study

**DOI:** 10.1186/s12909-026-09448-5

**Published:** 2026-06-01

**Authors:** Naziru Bashir Mukhtar, Khadija Muhammad Ali, Linzette Deidre Morris

**Affiliations:** 1 Department of Physiotherapy, Bayero University Kano, Kano, Nigeria; 2 Department of Physiotherapy, Maryam Abacha American University of Nigeria, Kano, Nigeria; 3 Department of Physiotherapy, Federal University of Health Sciences, Azare, Nigeria; 4 Department of Physiotherapy, Aminu Kano Teaching Hospital, Kano, Nigeria; 5 Department of Rehabilitation Sciences, College of Health Sciences, QU Health sector, Qatar University, Doha, Qatar

**Keywords:** Pain Neuroscience Education, Pain Neurophysiology Knowledge, Healthcare Education, Knowledge Assessment, Bayero University Kano, Nigeria, Africa, Chronic Pain

## Abstract

**Background:**

Pain Neurophysiology Knowledge (PNK) refers to the understanding of the biological and physiological mechanisms underlying the experience of pain. For healthcare students, a solid grasp of pain neurophysiology is essential for the effective future management of patients experiencing pain, particularly chronic pain. The aim of this study was to assess the level of PNK among healthcare undergraduate students studying Dentistry, Medicine, Nursing, Optometry, and Physiotherapy at Bayero University Kano (BUK), Nigeria.

**Methods:**

A cross-sectional study was conducted using the Revised Neurophysiology of Pain Questionnaire (RNPQ) to evaluate PNK among healthcare students at BUK. Whole population sampling was employed to recruit registered healthcare students across the various disciplines at BUK. Descriptive statistics (frequencies, percentages, and means) were used to characterize the sample. Differences in scores between the study disciplines and levels of study were analyzed using one-way ANOVA and independent t-tests (IBM SPSS version 23). One-way ANOVA was used to compare mean scores across multiple disciplines and clinical levels where more than two groups were present. Where comparisons involved only two groups, independent samples t-tests were applied. Significance level was set at *p* < 0.05.

**Results:**

A total of 500 students from the Physiotherapy, Nursing, Medicine, Dentistry, and Optometry programs participated. The majority of participants (66.2%) were aged 21–25 years, and 57.2% were male. Medical students represented the largest group (27.0%), and second-year clinical students across disciplines comprised 38% of the sample. The mean PNK score across participants from all disciplines was 6.31, indicating moderate knowledge. Statistically significant differences in PNK across clinical years were observed within the Medicine and Nursing programs (*p* < 0.05).

**Conclusion / implications:**

Healthcare students at BUK demonstrated average knowledge of pain neurophysiology. This finding suggests a gap in PNK within the study population, which warrants further investigation to better understand the underlying factors contributing to the moderate knowledge levels observed. Future research should explore potential influences such as curricular content, teaching strategies, clinical exposure, and interprofessional education experiences, as well as institutional and contextual factors that may shape students’ understanding of pain neurophysiology.

## Background

Globally, the average prevalence of unspecified chronic pain is estimated at approximately 31%, underscoring its significance as a widespread public health concern [1, 2]. While acute pain serves a vital protective function and is essential for survival, chronic or persistent pain is complex and multifactorial, typically arising from an interplay between biological, psychological and social mechanisms [1–3]. The economic burden of chronic pain is also significant, extending beyond the expected healthcare expenditures [2]. Indirect costs arise from reduced productivity, absenteeism, early retirement, and the loss of workforce participation [2]. In many settings, chronic pain also contributes to increased reliance on social welfare systems while simultaneously imposing the burden of unpaid care from relatives and friends, thereby straining families, societies and national economies [2, 4]. Collectively, these factors render chronic pain not only a major public health issue, but also a critical economic challenge that affects national development and sustainability [4].

Given the complexity of chronic pain, it is recommended that patients be referred to a multidisciplinary pain management team composed of diverse healthcare professionals e.g. physiotherapists, doctors, etc. with specialized expertise [4, 5]. Decisions regarding the management of chronic pain are multifaceted, often requiring integration of biomedical interventions with psychosocial strategies, patient education, and long-term rehabilitation approaches [1–3]. Healthcare professionals should be well-versed in the biopsychosocial model of health, particularly in its application to chronic pain, and are expected to guide patients by explaining the nature of their pain and providing evidence-based strategies for its management [4, 5]. Traditional pain education models commonly employed by healthcare professionals typically tend to emphasize anatomy, biomechanics and pathoanatomy, often at the expense of integrating contemporary pain science and biopsychosocial perspectives [4, 6–8]. However, these models often fail to address the complexities of chronic pain, including peripheral and central sensitization, immune and endocrine responses, and other factors implicated in persistent pain states [7, 8].

In response to the limitations of traditional pain education models, a new framework known as Pain Neuroscience Education (PNE) was introduced, aiming to provide a more structured and evidence-based approach to teaching patients the mechanisms and multidimensional aspects of pain, particularly chronic pain [7, 8]. PNE involves educating patients about the biological and physiological processes underlying pain and has increasingly gained popularity among physiotherapists and other healthcare professionals as an effective tool for chronic pain management [7, 8]. PNE typically involves educational sessions that explain how the nervous system interprets signals from tissues through mechanisms such as peripheral and central sensitization, synaptic activity, and brain processing. Patients are taught that neural activation, whether upregulated or downregulated, can modulate their pain experience. They learn that pain is not always a direct reflection of tissue damage, but rather a product of nervous system processing influenced by psychosocial factors [7, 8]. To better understand how individuals conceptualize the biological mechanisms underpinning their pain, Pain Neurophysiology Knowledge (PNK) is often assessed using a validated questionnaire, namely the Neurophysiology of Pain Questionnaire (NPQ) [9]. This measure reflects the extent to which patients grasp the science of pain and its underlying processes [9]. When paired with PNE, which teaches patients about how pain is generated and maintained, PNK assessment becomes both a preventive strategy in acute pain contexts and a therapeutic intervention for those with chronic pain [9].

Adequate PNK levels is however also essential for future healthcare graduates, as it fundamentally shapes their ability to understand and assess pain, communicate effectively with patients, and implement appropriate management strategies [9, 10]. Students should learn at an undergraduate level, that pain is not always a direct reflection of tissue damage, but rather a complex experience produced by the nervous system and influenced by biological, psychological, and social factors [10]. This knowledge equips students and eventually graduates to approach patients more holistically, addressing not only the physical aspects of pain but also the psychosocial contributors that can exacerbate or perpetuate it [11]. PNK helps clinicians implement evidence-based approaches such as PNE, graded exposure, and self-management strategies. Strengthening PNK among future graduates is therefore essential, as it not only enhances clinical reasoning and patient outcomes but also supports the shift from a purely biomedical model to a more comprehensive, biopsychosocial approach in contemporary healthcare.

Despite substantial advances in pain science over the past five decades [12], research exploring PNK levels among students and graduates of healthcare programs is still relatively limited. A few studies have begun to emerge in recent years. A study conducted in Pakistan in 2024 found that medical students demonstrated satisfactory levels of PNK, indicating that the curriculum they were exposed to, provided a reasonable foundation in modern pain science principles [11]. The authors suggested that early and structured inclusion of pain neurophysiology content may have contributed to students’ positive knowledge scores, highlighting the potential impact of well-designed educational strategies even in resource-constrained settings. In Saudi Arabia, final-year physiotherapy students demonstrated slightly higher PNK scores than their junior counterparts. This improvement was modest and did not reach clinical significance [13]. This suggests that although students may gain some additional understanding as they progress through the program, the curriculum may not be providing sufficient depth or reinforcement of pain science concepts to produce meaningful gains in knowledge. The findings highlighted a potential need for more structured, comprehensive, and vertically integrated pain education across the physiotherapy training continuum. A study from Spain comparing PNK among students in Physiotherapy, Medicine, and Human Nutrition reported that final-year students possessed higher levels of knowledge than first-year students, especially regarding biopsychosocial components of pain. Nonetheless, even physiotherapy students, who received more extensive pain-related training, still demonstrated notable gaps, with overall understanding remaining inadequate to support effective chronic pain management [14]. Although scarce, the available studies do provide valuable insights into how PNK levels vary across regions and healthcare professions, and collectively underscore the need for more comprehensive curricula at undergraduate level.

In Africa, however, few studies have explored PNK among healthcare students or professionals. One example is a study from South Africa, which assessed PNK among physiotherapy students and highlighted notable gaps in foundational pain science knowledge [15]. The scarcity of such research across Africa is striking, especially given the continent’s substantial burden of musculoskeletal and chronic pain conditions, variations in healthcare training frameworks, and limited access to specialized pain services [16]. However, due to the substantial diversity in healthcare training models, resource availability, and pain education exposure across African countries, evidence from one setting cannot be assumed to reflect the broader continent. This lack of region-specific data is particularly concerning in contexts where the burden of chronic pain is high and access to specialized pain services remains limited. There is therefore a critical need for studies that evaluate PNK within African healthcare training programs to inform curriculum development and ultimately improve patient care. In response to this gap, the present study aimed to assess PNK among undergraduate healthcare students at Bayero University Kano (BUK), a major university in Nigeria.

## Methods

### Study design and setting

This study employed a descriptive cross-sectional design to assess PNK among healthcare students at BUK, Nigeria. The study was conducted on the campus of BUK, situated in Northern Nigeria. Nigeria is the most populous country in Africa, with a highly diverse population spread across 36 states. Among these, Kano State stands out as the most densely populated, making it a significant hub for educational and healthcare initiatives [17]. BUK, a premier public university in Kano State, Nigeria, has a strong reputation for academic excellence, particularly in healthcare training programs, including medicine, nursing, and allied health professions. The university has recently been ranked third overall among Nigerian universities and holds the distinction of being the highest-ranking institution in Northern Nigeria [18, 19].

### Study population

The target population comprised clinical-level undergraduate students enrolled in Medicine, Physiotherapy, Nursing, Optometry, and Dentistry at BUK. All programs included three clinical years, with the exception of Physiotherapy, which consisted of two clinical levels.

### Sampling and sample size calculation

Whole population sampling was employed for participant recruitment. All students enrolled (*n* = 500) in the clinical phase within the relevant programs at BUK during the study period were considered eligible and were invited to participate in the study. As the intention was to include the entire accessible student population rather than select a subset, the study adopted a census approach of the available cohort. Given that the target population was finite, clearly defined, and fully accessible, a formal sample size calculation was not deemed necessary. Instead of estimating a representative sample, the study aimed to capture data from the complete student body to maximize response coverage and reduce sampling bias within the institutional context. Nevertheless, response rate considerations and potential non-response bias were acknowledged in the interpretation of findings. All 500 self-administered questionnaires distributed were returned, yielding a 100% response rate.

### Study procedure and data collection procedure

Data were collected at a single point in time using structured validated questionnaires. A total of 500 copies of the Revised Neurophysiology of Pain Questionnaire (RNPQ) were distributed to eligible healthcare students at BUK, Nigeria. Distribution was proportionate to departmental enrollment: 135 questionnaires to Medical students, 45 to Dental Surgery students, 120 each to Nursing and Optometry students, and 80 to Physiotherapy students. All clinical students enrolled in the clinical phase of the selected programs (Physiotherapy, Nursing, Medicine, Dentistry, and Optometry) were approached in their respective classes by KMA during scheduled lectures. Lecturers were briefed on the study objectives and subsequently granted access to their students. Informed consent was obtained from all participants prior to data collection. Printed copies of the RNPQ were then distributed and completed manually by students during the class session, with questionnaires collected immediately upon completion. The entire process required only a few minutes for each participant.

Pre-clinical students from all the programs were excluded. Although pain concepts may be introduced in pre-clinical years two and three, we recruited only students who had completed the entire pre-clinical stage. This ensured students had at least some exposure to pain neurophysiology and were entering the clinical phase, where they begin direct interactions with patients experiencing pain.

### Study instrument

PNK was assessed using the English version of the RNPQ [20]. The original NPQ was developed to evaluate how individuals conceptualize the biological mechanisms underpinning their pain and has demonstrated adequate psychometric properties among patients with chronic spinal pain [9]. The RNPQ was subsequently refined using Rasch analysis to improve the measurement properties and uni-dimensionality of the original tool, providing a more robust assessment of PNK [21, 22]. The RNPQ has also been used in previous studies to assess students’ knowledge of pain neurophysiology [13]. It consists of 12 statements related to pain neurophysiology with response options of “True,” “False,” or “Undecided.” Each correct response is awarded one point, while incorrect or undecided responses receive zero. The maximum possible score was 12, and the minimum was 0.

The RNPQ demonstrates excellent test–retest reliability (ICC > 0.90), indicating high stability when no change in knowledge has occurred. Internal consistency following Rasch refinement was acceptable in the original study [9], although cross-cultural adaptations have reported variable findings. Moderate internal consistency in the Dutch version (Cronbach’s α ≈ 0.62) [21] and lower values in the Turkish version [22] have been reported, suggesting possible item heterogeneity in some populations. In a cross‑validation study of the Turkish version of the RNPQ in patients with neck pain, Cronbach’s alpha was reported as 0.81 [23], indicating high internal consistency for that version of the questionnaire. Other adaptations of the RNPQ have shown more modest internal consistency estimates in different samples. For example, a Turkish physiotherapy student sample reported a Cronbach’s alpha of 0.57 for the RNPQ‑TR [24]. These values illustrate that internal consistency of the RNPQ can vary by language version and sample, but commonly reported Cronbach’s alpha coefficients range from moderate (0.57) to higher reliability (0.81). Construct validity is supported by the instrument’s ability to differentiate between groups with expected differences in pain knowledge (e.g., clinicians versus patients) [21, 22]. While translated versions indicate that the RNPQ can be successfully adapted, careful cultural validation is recommended prior to use.

### Data analysis

The data collected was analyzed using IBM SPSS version 23. Descriptive analysis of mean, frequencies and percentages were used to summarize the data. One-way analysis of the variance (ANOVA) and independent sample t-tests were used to test for difference in scores across different healthcare students’ characteristics. The descriptive variables analyzed included gender, age, level of clinical study, program of study, whether pain was taught, and the specific courses in which pain content was delivered. These variables were summarized using descriptive statistics. To examine differences in mean PNK scores, One‑way ANOVA was applied to compare scores across the five programs and, within programs offering three levels of clinical training (clinical year 1, clinical year 2, and clinical year 3), to assess differences between levels. Where significant differences were identified, post‑hoc analyses were conducted. For programs with only two levels of clinical training (clinical year 1 and clinical year 2), independent t‑tests were used to compare mean scores.

## Results

### Sociodemographic characteristics

A total of 500 participants were included in the study. Table 1 presents the sociodemographic profile of the participants. The majority (66.2%) were aged 21–25 years, followed by 30.0% aged 26–30 years. Gender distribution showed a predominance of male participants (57.2%). Medical students represented the largest group (27.0%), followed by Nursing and Optometry students (24.0% each), Physiotherapy students (16.0%), and Dental students (9.0%). Regarding academic level, 38.0% of participants were in their second clinical year, 35.0% in their first clinical year, and 27.0% in their third clinical year. A substantial majority (93.6%) of students recalled being taught about pain. Among these, 57.6% participants reported receiving pain-related education in neurophysiology courses. Additional details are presented in Table 1.

**Table 1:** Showing socio-demographic characteristics of the participants

Variables	Frequency	Percentage
AGE (YEARS)		
16–20	7	1.4
21–25	331	66.2
26–30	150	30.2
≥ 31	12	2.4
GENDER		
Male	286	57.2
Female	214	42.8
ACADEMIC PROGRAM		
Medicine	135	27.0
Dentistry	45	9.0
Nursing	120	24.0
Optometry	120	24.0
Physiotherapy	80	16.0
CLINICAL YEAR		
First clinical year	175	35.0
Second clinical year	190	38.0
Third clinical year	135	27.0
PAIN CONTENT DELIVERED IN PROGRAM		
Yes	468	93.6
No	32	6.4
SPECIFIC COURSE IN WHICH PAIN CONTENT WAS DELIVERED		
None	32	6.4
Pain management	5	1%
Maternal and child care	6	1.2%
Ocular pathology	6	1.2
Pathology/Pathophysiology/Histopathology	11	2.2%
Massage Therapy	9	%
Neuroanatomy	10	2.0
Medical rehabilitation	12	2.4
Pharmacology	15	3.0
Anesthesia	20	4.0
Medical surgical nursing	58	11.6
Neurophysiology	288	57.6
Other i.e. paediatrics, exercise therapy, psychology, cryotherapy, etc.	28	5.6%

### Participants knowledge about pain neurophysiology

The participants’ knowledge about pain neurophysiology was assessed using the RNPQ. Their mean (SD) scores for knowledge about pain neurophysiology is presented in the table below (Table 2) for each healthcare discipline.

**Table 2:** Differences in pain neurophysiology knowledge between academic programs

Academic program	Mean	SD	df	F	*p*
Medicine	6.41	1.850	4	0.837	0.502
Dentistry	6.44	1.829			
Nursing	6.03	2.165			
Optometry	6.39	1.950			
Physiotherapy	6.39	1.739			
Overall	**6.31**	**1.93**			

*SD* Standard deviation, *d.f* degree of freedom, *F* Overall F-statistic, *p* Significant value

### Differences in pain neurophysiology knowledge between academic programs

One-way ANOVA was used to test the differences in mean PNK scores between academic programs of the participants. A test of homogeneity of variances was conducted and the assumption was met (*p* > 0.05), indicating suitability for ANOVA. Table 2 shows that there were no significant differences in PNK scores between academic programs (*p =* 0.502).

### Differences in pain neurophysiology knowledge between clinical years within the programs

Table 3 presents the differences in mean PNK scores across clinical years for all programs, with the exception of Physiotherapy. A one-way ANOVA was conducted to examine whether knowledge levels differed between clinical years within each program. Overall, no statistically significant differences were observed between clinical years in most programs, suggesting that PNK remained relatively stable as students progressed through training. However, significant differences (*p* < 0.05) were observed between the clinical years in the Medicine and Nursing programs. In the Medicine program, post-hoc analysis revealed a significant decline in PNK from the first clinical year to the second clinical year. In contrast, in the Nursing program, there was a significant improvement in knowledge over the same period, with students in the second clinical year demonstrating higher mean scores compared to those in the first clinical year.

**Table 3:** Differences in pain neurophysiology knowledge between clinical years within the programs

	Mean	SD	df	F	*p*
Medicine					
Clinical year 1	7.18	1.448	2	5.743	0.004*
Clinical year 2	5.93	1.961			
Clinical year 3	6.30	1.843			
Dentistry					
Clinical year 1	7.07	2.282	2	1.343	0.272
Clinical year 2	6.07	1.163			
Clinical year 3	6.20	1.821			
Nursing					
Clinical year 1	5.13	1.727	2	7.933	0.001*
Clinical year 2	6.95	1.921			
Clinical year 3	6.02	2.434			
Optometry					
Clinical year 1	6.38	1.764	2	0.060	0.941
Clinical year 2	6.48	1.961			
Clinical year 3	6.33	2.153			

Clinical year (A)	Clinical year (B)		Mean Difference (A-B)		*p*
Medicine Post-hoc analysis					
Clinical year 1	Clinical year 2		1.25		0.003*
	Clinical year 3		0.88		0.077
Clinical year 2	Clinical year 1		-1.25		0.003*
	Clinical year 3		-0.37		0.576
Clinical year 3	Clinical year 1		-0.88		0.077
	Clinical year 3		0.37		0.576
Nursing Post-hoc analysis					
Clinical year 1	Clinical year 2		-1.83		0.000*
	Clinical year 3		-0.90		0.126
Clinical year 2	Clinical year 1		1.83		0.000*
	Clinical year 3		0.93		0.112
Clinical year 3	Clinical year 1		0.90		0.126
	Clinical year 2		-0.93		0.112

*SD* Standard deviation, *d.f* degree of freedom, *F* Overall F-statistic, *p* Significant value

*= Significant

For the Physiotherapy program, which included only two clinical years, an independent t-test was used instead to assess differences between clinical levels. No statistically significant differences in PNK scores were observed between clinical levels among Physiotherapy students (*p* = 0.507).

## Discussion

This study aimed to evaluate PNK among healthcare students at BUK, with a focus on variations across academic programs and clinical years. Overall, the participants included in this study demonstrated a moderate level of PNK. For healthcare students, possessing only an average understanding of pain neurophysiology may be inadequate and could compromise the effectiveness of patient management, especially for individuals experiencing chronic pain.

No statistically significant differences were observed in RNPQ scores across sociodemographic variables such as age, sex, clinical year, or academic program. This uniformity may be due to the fact that all study participants were drawn from health sciences programs within the same institution and were exposed to broadly similar foundational curricula in pain education and related biomedical sciences. This may have contributed to relatively homogeneous levels of PNK across groups. While this consistency ensures a baseline knowledge of human biology, it may inadvertently result in limited exposure to advanced or clinically relevant pain neuroscience concepts tailored to each program, as confirmed by previous studies [11, 13–15]. In addition, clinical year progression in these programs may not necessarily correspond to structured or incremental formal teaching in pain neurophysiology, which could further explain the lack of between-group differences. Consequently, students across different academic programs may finish their pre-clinical training with comparable but only average PNK, without the necessary reinforcement and application during clinical years.

The majority of participants reported receiving instruction on pain during their training, with 57.6% recalling lessons in neurophysiology, a course typically offered at the pre-clinical level. While early exposure to pain concepts is valuable, the absence of structured pain education during clinical years is a concern. The only clinical course with notable recall was Medical-Surgical Nursing. which is specific to the Nursing program. This pattern observed in this study indicates that pain education at BUK may largely be pre-clinical. According to previous literature, early exposure to neurophysiology may provide a theoretical foundation, but without ongoing, program-specific reinforcement during clinical training, students may struggle to apply pain knowledge effectively in patient care [14, 15]. Further research is warranted to better understand the structure and timing of pain education within the curricula at BUK and how this may potentially influence students’ ability to translate theoretical knowledge into clinical practice – at undergraduate and postgraduate level.

The mean level of PNK (6.31) observed in this study is consistent with findings from similar research across different regions and health disciplines. For example, physiotherapy students in Saudi Arabia achieved a mean score of 6.2 (55.8%) on the 12-item RNPQ, indicating an overall average level of knowledge [13]. Likewise, final-year physiotherapy students in South Africa scored 58%, which also falls within the average knowledge range [15]. Although Shahzad et al. [11] reported high levels of knowledge among Pakistani healthcare students, their study did not define an “average” category; under the classification used in the present study, their mean score of 7.0 would be considered average. Similarly, Adillón et al. [14] found that final-year Medicine and Physiotherapy students in Spain demonstrated an overall average understanding of pain neuroscience concepts. Collectively, these findings suggest that across diverse educational settings and health professions, undergraduate students generally exhibit moderate knowledge of pain neurophysiology, highlighting the ongoing need for more comprehensive pain neurophysiology in health curricula to ensure effective management of chronic pain and its complexity at a postgraduate level. This consistent pattern across various contexts likely reflects a systemic global gap in undergraduate pain education rather than a country-specific weakness [25–27]. Pain remains one of the leading causes of disability worldwide, yet pre-licensure training often allocates limited time to contemporary pain science, particularly the biopsychosocial and nociplastic dimensions of persistent pain [28]. When moderate knowledge levels are observed across high-, middle-, and low-income settings alike, this convergence points to structural curricular limitations, insufficient integration of modern pain frameworks [25–27], and limited reinforcement during clinical training. However, the implications of this gap are not uniform across contexts. In Nigeria and many African countries, the consequences may be amplified by health system constraints [29]. Chronic pain, particularly low back pain and other musculoskeletal disorders [16, 30], contributes substantially to disability, work absenteeism, and reduced productivity. At the same time, access to specialist pain services, multidisciplinary clinics, and advanced diagnostics remains limited [31]. As a result, frontline providers, including general physicians, nurses, and physiotherapists, bear primary responsibility for pain management.

Significant differences in PNK were observed among clinical years of Medical and Nursing students. Among Medical students, first-year clinical students scored higher than their second-year counterparts, possibly due to the recency of their neurophysiology coursework. Conversely, second-year Nursing students scored higher than first-years, likely reflecting exposure to pain-related content in Medical-Surgical Nursing. Differences in clinical experiences among Nursing, Medicine, and allied health programs could however offer opportunities to contextualize pain education, yet the absence of structured, longitudinal integration may prevent students from developing the deeper understanding required for patient-centered care, particularly in chronic pain management. Moreover, the lack of significant variation by demographic factors suggests that deficits in PNK are systemic rather than isolated to specific student groups. This highlights the potential value of curriculum reform that emphasizes not only the quantity but also the clinical relevance of pain education, including interprofessional learning opportunities that mirror real-world healthcare environments. Strengthening these aspects could improve students’ readiness to educate patients and contribute meaningfully to multidisciplinary pain management teams [5].

### Study limitations

Despite employing whole-population sampling of healthcare students at BUK in Nigeria, several limitations related to this study warrant consideration. First, the study was conducted in a single university setting, which may limit the generalizability of the findings to other institutions within Nigeria, across Africa or globally. Second, the cross-sectional design further limits causal inference, as associations observed cannot establish temporal or directional relationships. Third, conducting the survey during class time may have introduced response bias, as students could have felt rushed or influenced by the classroom environment. Fourth, the psychometric properties of the RNPQ were not evaluated in this sample, as this was beyond the study objectives, which focused on overall outcomes. While the RNPQ has demonstrated acceptable properties in other populations, the lack of sample-specific validation may limit interpretability and generalizability. Future studies should assess its reliability, validity, and responsiveness in similar populations, including item-level and factor structure analyses. Additionally, reliance on self-reported data regarding the courses in which pain was taught raises the possibility of recall bias. The assessment focused exclusively on PNK, without evaluating practical application or long-term retention, thereby limiting insight into clinical competence. We further acknowledge that direct comparisons with Western studies should be interpreted cautiously due to differences in healthcare education systems, curricula, cultural contexts, and assessment approaches. These comparisons were intended to contextualize our findings within the broader literature rather than suggest equivalence, recognizing that variations in educational frameworks and pain education exposure may limit comparability. Finally, given that Nigeria as a low-to-middle-income country with limited specialized pain infrastructure, structural and educational disparities may also affect the students’ exposure to pain neuroscience content, further constraining the broader applicability of the results.

## Conclusion

This study revealed that healthcare students at BUK possess an average level of knowledge regarding pain neurophysiology. This finding suggests a gap in PNK within the study population, which warrants further investigation to better understand the underlying factors contributing to the moderate knowledge levels observed. Future research should explore potential influences such as curricular content, teaching strategies, clinical exposure, and interprofessional education experiences, as well as institutional and contextual factors that may shape students’ understanding of pain neurophysiology. Such insights would be valuable in informing targeted educational interventions to strengthen PNK among healthcare students.

This study provides a broad multi-professional perspective on PNK in one large African university, offering preliminary insights that may be directly relevant to health professions education in Africa and globally. Further investigation is warranted to better delineate the strengths and gaps in students’ understanding of pain neurophysiology and to inform curriculum developers, educators, and policymakers on the need for targeted enhancement of pain education, particularly during the pre-clinical and clinical training years.

## Data Availability

All data and materials can be requested from the first author.

## Abbreviations

BUK: Bayero University Kano
PNK: Pain Neurophysiology Knowledge
NPQ: Neurophysiology of Pain Questionnaire
RNPQ: Revised Neurophysiology of Pain Questionnaire
PNE: Pain Neuroscience Education
